# Public attitudes to a human challenge study with SARS-CoV-2: a mixed-methods study

**DOI:** 10.12688/wellcomeopenres.17516.1

**Published:** 2022-02-10

**Authors:** Caroline Barker, Katharine Collet, Diane Gbesemete, Maria Piggin, Daniella Watson, Philippa Pristerà, Wendy Lawerence, Emma Smith, Michael Bahrami-Hessari, Halle Johnson, Katherine Baker, Ambar Qavi, Carmel McGrath, Christopher Chiu, Robert C. Read, Helen Ward

**Affiliations:** 1 NIHR Southampton Clinical Research Facility and NIHR Southampton Biomedical Research Centre, University Hospital Southampton NHS Foundation Trust, Southampton, SO16 6YD, UK; 2 NIHR Applied Research Collaboration Wessex, University Hospital Southampton NHS Foundation Trust, Southampton, SO16 6YD, UK, UK; 3 NIHR Imperial Biomedical Research Centre, Patient Experience Research Centre, Imperial College London, London, W2 1NY, UK; 4 School of Clinical and Experimental Sciences, Faculty of Medicine and Institute for Life Sciences, University of Southampton, Southampton, S016 6YD, UK; 5 MRC Lifecourse Epidemiology Unit, University of Southampton, Southampton, S016 6YD, UK; 6 Global Health Research Institute, School of Human Development and Health, Faculty of Medicine, University of Southampton, Southampton, SO17 1BJ, UK; 7 National Heart and Lung Institute, Imperial College London, London, W2 1NY, UK; 8 Department of Infectious Diseases, Imperial College London, London, W2 1NY, UK

**Keywords:** Ethics, human challenge study, consultation, public, acceptability, COVID-19

## Abstract

**Background:** Human challenge studies involve the deliberate exposure of healthy volunteers to an infectious micro-organism in a highly controlled and monitored way. They are used to understand infectious diseases and have contributed to the development of vaccines. In early 2020, the UK started exploring the feasibility of establishing a human challenge study with SARS-CoV-2. Given the significant public interest and the complexity of the potential risks and benefits, it is vital that public views are considered in the design and approval of any such study and that investigators and ethics boards remain accountable to the public.

**Methods:** Mixed methods study comprising online surveys conducted with 2,441 UK adults and in-depth virtual focus groups with 57 UK adults during October 2020 to explore the public’s attitudes to a human challenge study with SARS-CoV-2 taking place in the UK.

**Results:** There was overall agreement across the surveys and focus groups that a human challenge study with SARS-CoV-2 should take place in the UK. Transparency of information, trust and the necessity to provide clear information on potential risks to study human challenge study participants were important. The perceived risks of taking part included the risk of developing long-term effects from COVID, impact on personal commitments and mental health implications of isolation. There were a number of practical realities to taking part that would influence a volunteer’s ability to participate (e.g. Wi-Fi, access to exercise, outside space and work, family and pet commitments).

**Conclusions:** The results identified practical considerations for teams designing human challenge studies. Recommendations were grouped: 1) messaging to potential study participants, 2) review of the protocol and organisation of the study, and 3) more broadly, making the study more inclusive and relevant. This study highlights the value of public consultation in research, particularly in fields attracting public interest and scrutiny **.**

## Introduction

The COVID-19 pandemic has had a devastating impact on health, society, and the economy at a global level. The search for an “exit strategy” has resulted in an unprecedented level of scientific research, moving at extraordinary pace. The public have more interest in clinical and epidemiological research than ever before, with hundreds of thousands volunteering globally ^1, 2^. With this urgency to gain understanding and develop effective vaccines and treatments, the potential use of human challenge with SARS-CoV-2 has been discussed among ethicists, scientists, and the World Health Organisation (WHO) ^3– 8^.

Human challenge, or controlled human infection, is a clinical research methodology involving the deliberate exposure of human volunteers to specified infectious micro-organisms in a highly controlled and monitored way. This allows detailed study of the specific infection from the point of exposure, providing valuable insight into the interactions between micro-organism and host. This type of study also enables the quick and accurate assessment of the impact of protective and therapeutic interventions ^9, 10^. Human challenge is an increasing area of research and has contributed towards the understanding of many different pathogens and to the development of vaccines ^11, 12^. The ethical implications of human challenge have been considered in detail and practical and regulatory guidelines are in place ^13– 15^. However, the concept and benefits of human challenge may not be widely known, or immediately understandable, outside of the academic community.

Early in the course of the COVID-19 pandemic, the possible utility of a human challenge model with SARS-CoV-2 (the causative agent of COVID-19) was suggested, particularly in expediting the efficacy assessment of vaccine candidates ^4^. Such a model could have the potential to test multiple vaccine candidates or therapeutic agents to rapidly identify the most promising, requiring only small numbers of healthy volunteers without relying on community transmission. It could also allow detailed immunological investigation of potential correlates of protection, duration of immunity and the potential for re-infection ^6, 16^. Such a model could therefore have very high scientific and societal value by significantly reducing the health, social and economic impacts of the pandemic.

However, concerns have been raised about the ethical acceptability of such a study, particularly about the level of risk to individual volunteers in the absence of an effective rescue treatment. The incomplete understanding of the risk factors associated with severe COVID-19 disease and the potential longer-term outcomes mean that it is not yet possible to fully quantify or confidently communicate the absolute risk to volunteers, nor to completely mitigate it. The potential of human challenge studies to significantly accelerate the availability of a universally effective vaccine has also been questioned, both in terms of the time that could be saved in comparison to field studies, and the generalisability of results to the broader population. A further concern raised is the potential damage to human challenge studies in particular, and clinical research in general, if a single negative outcome were to occur in such a high-profile study ^3, 6, 16– 18^.

In May 2020, the WHO released criteria for the ethical acceptability of a human challenge study with SARS-CoV-2 taking place. One criterion included the involvement of the public, to take into account their viewpoints and concerns, and to ensure that any such study is fully transparent, providing complete, easily understandable information to potential participants and to the community ^7^.

It is vital that researchers are accountable to the public. The purpose, risks and benefits of proposed research must be communicated adequately, allowing public concerns and opinions to be considered, and, where appropriate, to shape the design of studies. Maintaining public trust is necessary to ensure that research outputs are widely accepted and therefore can be translated into real-world impacts.

In April 2020, a public consultation was carried out on the possibility of a human challenge study with SARS-CoV-2 in the UK. Focus groups of 18 to 40-year olds were given a brief explanation of human challenge, and opinions were gathered about acceptability and perceived risks and benefits. The responses were positive overall, but with evidence that further information would be required to answer a variety of concerns and questions ^19^.

We built on this initial work by undertaking a broader consultation to explore public understanding of the concept of a human challenge study in general and the acceptability of a human challenge study with SARS-CoV-2 taking place in the UK. Specific concerns and opinions raised by the first public consultation in April 2020 were explored in greater depth. This work was completed prior to the approval of the current COVID-19 vaccines which may have changed the perceived balance of risks and benefits. However, a human challenge model still has the potential to further the immunological and pathophysiological understanding of SARS-CoV-2 infection and to facilitate both the optimisation of current vaccine programs and the evaluation of the next generation of vaccines. The opinions and concerns that were gathered were used to inform the study design and Research Ethics Committee’s consideration of the first SARS-CoV-2 human challenge study which received regulatory approval in the UK in February 2021 ^20^.

## Methods

### Study design

This study used a mixed methods approach with online surveys and focus groups.

The online survey was based on the scoping work undertaken and published by the University of Southampton in April 2020 ^19^ exploring public perceptions of human challenges studies with coronavirus (COVID-19). It was first designed by the research study team then tested with members of the public (see patient and public involvement section). Recruitment was done via two methods, described below, and quality checked by both the YouGov and Imperial teams. The same core questions were used in both the samples. The survey questions are available in the *Extended data* ^21^.

The survey included multiple-choice and free text questions and took approximately 20 minutes to complete. Following consent, respondents were asked to watch an animation explaining the concept of a human challenge study with coronavirus ( https://www.youtube.com/watch?v=FncT4ki-Uww). Questions were grouped under the following sections: (1) current knowledge of human challenge studies, (2) perspectives on human challenge studies in general, (3) perspectives on taking part in a human challenge study, for oneself, someone close to them, and employees, where relevant, (4) background and demographic questions.

Focus groups were chosen as a method of gaining a variety of views and experiences from people of different genders, ages and to capture group dynamics ^22, 23^. A semi-structured discussion guide was designed to stimulate discussion on the acceptability and conditions of the human challenge study, including recruitment, screening, isolation and effects on family and employers. The guide was based on the themes identified in the surveys, previous public involvement work and further developed by the research study team.

At least 24 hours in advance of the focus groups, discussants were sent the following link for further information about human challenge studies with coronavirus ( https://www.hic-vac.org/public-information/human-challenge-studies-coronavirus-towards-effective-vaccines). Focus groups were conducted on Zoom Pro. Each online session began with an introduction by a researcher, experienced in working on human challenge studies, who provided a concise summary on why a human challenge study with coronavirus is being considered in the UK and what it might involve. The researcher then answered participants’ questions. One researcher allocated participants into virtual breakout rooms by age group for the focus group discussions (18–30 years or 31+ years). Participants were divided by age group to ask more specific questions directed to those who were eligible (in age) to volunteer in a human challenge study, and then those who might be a relative, friend or employer of a volunteer. Each breakout room was led by one experienced qualitative facilitator, supported by an observer who took detailed notes. Participants were reimbursed £30 for their participation in a focus group.

All focus group discussion audio-recordings were transcribed. The observers cleaned the transcripts and checked the accuracy against their observation notes.

### Sampling

The survey sampled adults (aged 18 and over) living in the UK using two methods: 1) a cross-sectional non-random sample of the UK population (N=2,137) via an online omnibus survey disseminated by YouGov to their volunteer panel between 8 and 9 October 2020; and 2) a convenience sample of the general UK population (N=304) via an online survey hosted on Qualtrics and disseminated by the research team through established public partner and community networks, VOICE-global online involvement platform, and social media between 8 and 13 October 2020.

Following the survey, adults aged 18 and over were then invited by email to online focus groups carried out between 15 and 21 October 2020 that explored the public’s attitudes in more depth. This convenience sample (N=57) were recruited either from: 1) those who completed the Qualtrics survey and signed up to hear about future opportunities relating to the study; or 2) established channels, public partners and community networks at Imperial College, London and the University of Southampton.

### Data analysis

Descriptive analysis was used to describe the public’s (quantitative) responses in the survey and assess the frequency, proportions and demographics of the attitudes and opinions shared. Descriptive statistics for all variables present the number of respondents and the weighted percentages (AQ, CB, KC). For quantitative analysis, datasets from both YouGov and the Qualtrics samples were merged and analysed using Microsoft Excel.

Focus group transcripts were read by members of the research team (DG, DW, HJ, KB, PP, WL). Initial codes were identified to classify the data, and then organised into themes to answer the research question “What do you think about there being a coronavirus human challenge study conducted in the UK?”. Using inductive coding, the research team developed a coding framework to represent the themes and sub-themes. The transcripts were coded against the coding framework using NVivo software version 12. This involved double-coding to inform iterations to the coding framework until all were satisfied that it provided a coherent framework for answering the research question. Thematic analysis was chosen because it provides detailed and rich accounts of complex data in a flexible manner, yet has rigorous methods to enhance objectivity ^24^. COREQ guidance was used to structure reporting ^25^.

The free text responses from the survey were coded separately in NVivo and reviewed against both the survey responses and the focus group coding framework (KC, PP). All were satisfied that these themes were sufficient to capture the views in the survey. Free text results have been incorporated to bolster analysis for both qualitative and quantitative analyses.

### Patient and public involvement

A group of 28 people attended a 1.5 hour virtual discussion on Zoom to review aspects of the study design for this public consultation. Individuals were invited through existing patient and public involvement networks at Imperial College, London and the University of Southampton and by advertising on the VOICE-global online involvement platform.

Participants were shown the animation and asked to complete the draft survey. Break out room discussions focused on suggestions to improve the animation and the survey. Following the meeting, three public contributors were invited to review the draft website text for focus group attendees. Subsequent changes were made to the animation, survey questions and website text. The activity also provided the research team with an idea of how long completion of the survey would take, to include in the participant information sheet.

### Ethics approval

The research aspects of this study were granted ethics approval by Imperial College London Research Ethics Committee (ICREC reference: 20IC6319). Participants were provided with an information sheet and provided written consent. Patient and public involvement exercises did not require ethical activity due to the nature of the activity. 

## Results

A total of 2,441 respondents completed the survey (8 ^th^-13 ^th^ October 2020) across the two survey data collection methods. Details of age, gender and ethnicity are shown in Table 1. Those who completed the Qualtrics survey were asked if they were willing to receive an invitation to attend a focus group. Subsequently 57 discussants attended one of nine focus groups (15 ^th^-21 ^st^ October 2020). Focus group discussions were split into those who are typically eligible, by age, for a human challenge study (18–30 years) and those who were not, to ensure only relevant topics were explored with discussants. Details by age, gender and ethnicity are reported in Table 2. The focus group discussions lasted between 59 and 80 minutes (mean = 65 minutes).

**Table 1.** Survey respondent characteristics.

Variable	Category	N	%	Variable	Category	N	%
Age	18–24	136	5.6	Gender	Male	1161	47.6
	25–34	355	14.5		Female	1273	52.2
	35–44	420	17.2		Non-binary/Gender variant	1	0.0
	45–54	450	18.4		Not listed	1	0.0
	55+	1064	43.6		Prefer not to say	2	0.1
	No answer	16	0.7		No answer	3	0.1
Variable	Category					N	%
Ethnicity	White						
	English/Welsh/Scottish/Northern Irish/British					2153	88.2
	Irish					29	1.2
	Gypsy or Irish Traveller					2	0.1
	Any other White background					106	4.3
	Mixed/Multiple ethnic groups						
	White and Black Caribbean					9	0.4
	White and Black African					2	0.1
	White and Asian					13	0.5
	Any other Mixed/Multiple ethnic background					20	0.8
	Asian/Asian British						
	Indian					29	1.2
	Pakistani					11	0.5
	Bangladeshi					8	0.3
	Chinese					7	0.3
	any other Asian background					8	0.3
	Black/African/Caribbean/Black British						
	African					13	0.5
	Caribbean					3	0.1
	any other Black/African/Caribbean background					3	0.1
	Other ethnic group						
	Arab					1	0.0
	Any other ethnic group					4	0.2
	Prefer not to say					17	0.7
	No answer					3	0.1

**Table 2.** Focus group discussant characteristics.

Variable	Category	N	%	Variable	Category	N	%
Age	18–30	26	45.6	Gender	Male	29	50.8
	31+ years	31	54.4		Female	27	47.4
					Non-binary/Gender variant	1	1.8
Variable	Category					N	%
Ethnicity	White						
	English/Welsh/Scottish/Northern Irish/British					33	57.9
	Irish					2	3.5
	Gypsy or Irish Traveller					0	0
	Any other White background					6	10.5
	Mixed/Multiple ethnic groups						
	White and Black Caribbean					0	0
	White and Black African					1	1.8
	White and Asian					1	1.8
	Any other Mixed/Multiple ethnic background					0	0
	Asian/Asian British						
	Indian					5	8.8
	Pakistani					4	7.0
	Bangladeshi					1	1.8
	Chinese					0	0
	any other Asian background					2	3.5
	Black/African/Caribbean/Black British						
	African					0	0
	Caribbean					1	1.8
	any other Black/African/Caribbean background					0	0
	Other ethnic group						
	Arab					1	1.8
	Any other ethnic group					0	0

**Figure 1.**
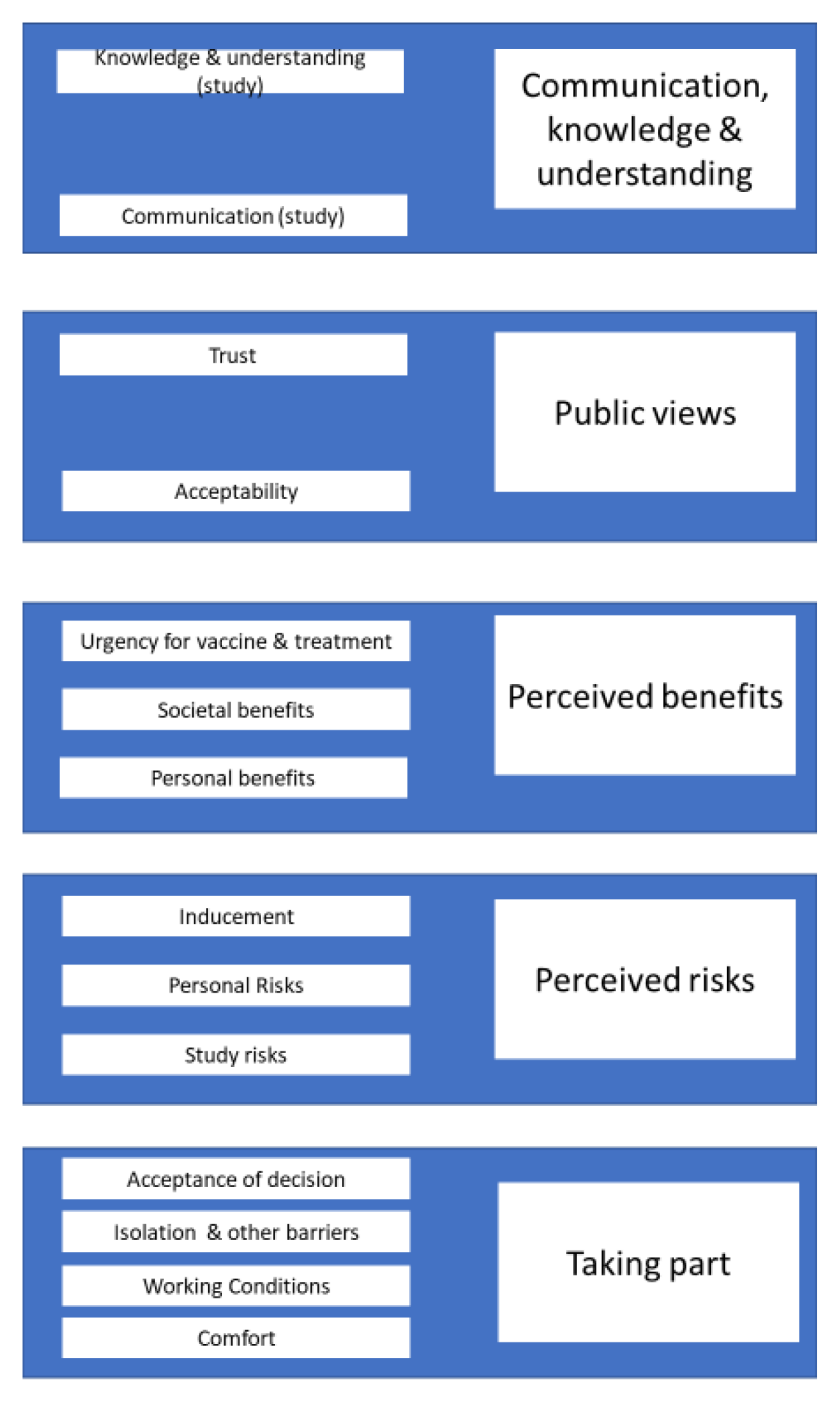
Thematic map. The following thematic map provides a visual overview of all results, both survey and FGD, as conceptualized in the methods section of the paper.

Five themes were identified from the survey data and thematic analysis of focus groups and are outlined below. Each theme is illustrated using a combination of descriptive statistics from the survey and quotes from the focus groups and survey free text responses (see Figure 1 for visual representation).

### 1. Communication, knowledge and understanding of human challenge studies

This theme captures people’s knowledge and understanding of human challenge studies. It includes what people identified to be important aspects of communication in relation to such studies. Focus group discussants reflected on the information they had received from the consultation team.

***Knowledge and understanding of human challenge studies***

Before taking part in the survey, respondents were asked whether they had ‘read, seen or heard anything about the idea of a human challenge study with coronavirus (COVID-19)’. A total of 78% of survey respondents had no awareness of human challenge studies with coronavirus ( Table 3a).

**Table 3.** Existing awareness of the idea of a human challenge study with coronavirus (a) and sources of information (b). Percentages for sources of information are presented as a percentage of the number of people who had previously read, seen or heard about the idea of a human challenge study with coronavirus.

(a)		(b)	
Had respondents read, seen or heard anything about the idea of a human challenge study with coronavirus (COVID-19)?		Sources of information	Percentage (Count)
**Answer**	**Percentage (Count)**	News or media coverage	76.0% (323)
Yes	17.4% (425)	Social media	23.3% (99)
No	78.0% (1903)	Personal conversation	14.4% (61)
Don't know	4.2% (102)	In depth or scientific articles	19.1% (81)
Prefer not to say	0.4% (9)	Other	15.3% (65)
Unanswered	0.1% (2)	Don't know/ can't recall	3.1% (13)

Focus group discussants were asked what they knew about human challenge studies, including how they might differ from other studies. A range of knowns and unknowns, as well as common misperceptions, were identified. Although questions relating to public understanding were not directly asked in the survey, these themes were also identified in the open text responses.

*“You've mentioned earlier that it would be a controlled environment. Does that mean that the food that they're eating … the water that they will be drinking are all the same?” [FG8_18–30]*
*“If this is a standard part of testing a vaccine, I presume it’s done under conditions which are as safe as possible and with full understanding of the risks involved on the part of the volunteers” [Survey respondent]*

***Communication about the study***

Of the 17% of survey respondents who had an existing awareness of human challenge studies, the most common source of information was news or media coverage (76%) ( Table 3b). Focus group discussants described how useful they had found the different types of information they had been given during the consultation.

*“I thought that introduction was really helpful. I'd also found the video [the animation] extremely helpful.” [FG6_31+] *
*“I thought the questions [answered as part of the focus group introduction] were quite good… I think those questions will be quite common for people just in general” [FG7_18–30] *

Participants reflected on the best ways to communicate with the general public about a human challenge study, and what specifically needed to be communicated. This included ideas of transparency, trust and clear information on risks.

*“I think you need that transparency, because you need people on side … to understand what's happening, understand what's been done to try and resolve it … there's been so much misinformation that I think people already at a pretty dangerous point with the way that they see things”. [FG5_31+] *
*“We live in an age where information can be altered and changed and cannot trust what we read so to be able to kind of confidently go to a source, to be able to go to a website and be able to understand exactly why this trial is going ahead and the acknowledgment of the risk”. [FG7_18–30]*

**Figure 2.**
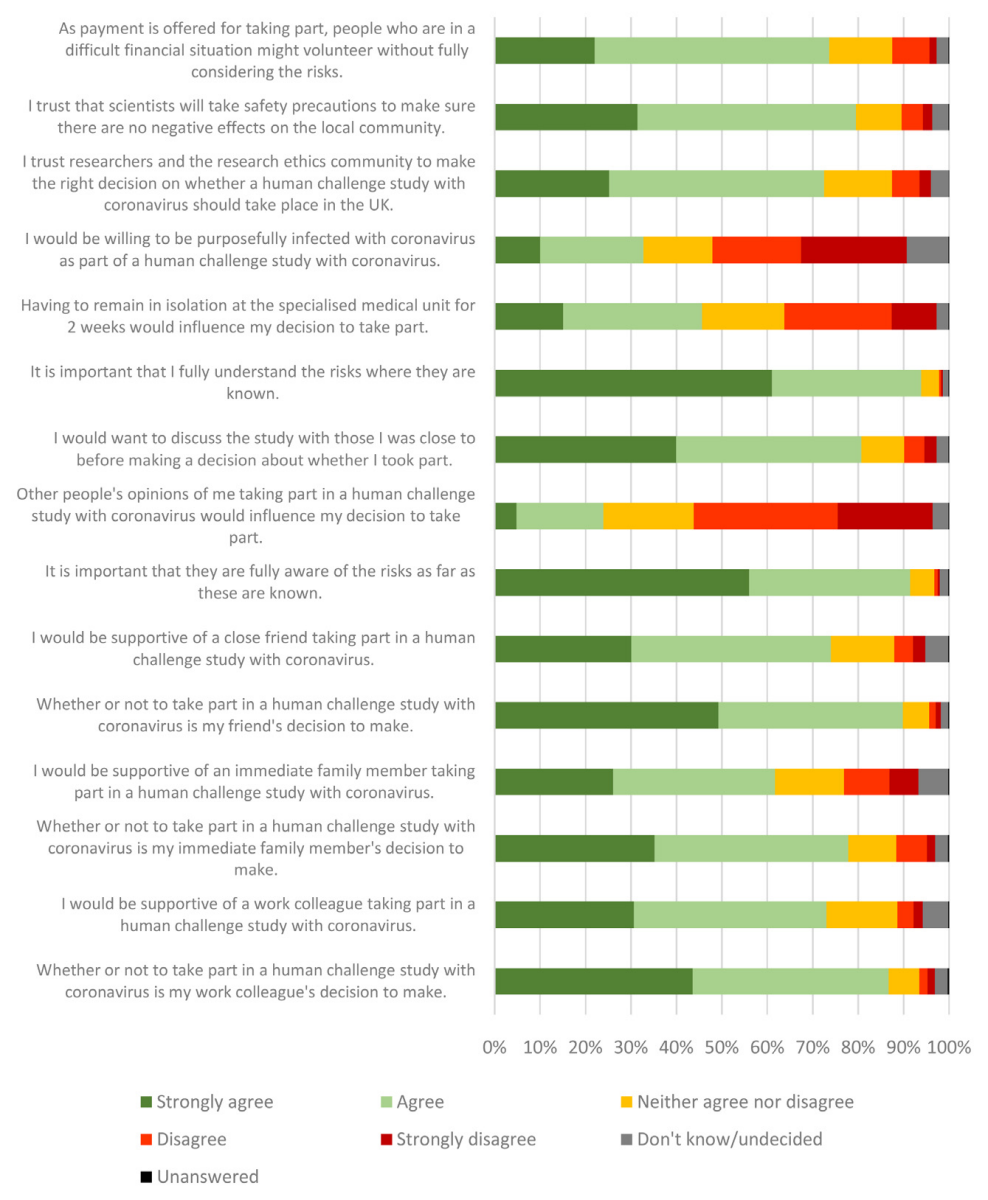
Opinion statements relating to a human challenge study with coronavirus. Various opinion statements were asked of participants throughout the survey to better understand the public’s views on HCS with coronavirus. Selected statements are included in the chart below.

In line with discussions relating to clear information on risks, survey respondents were asked to rate how strongly they agree or disagree with statements relating to understanding of risk ( Figure 2). When considering their own participation in a human challenge study (regardless of whether they would be eligible to take part), 94% of respondents strongly agreed or agreed that it was important they fully understood the risks where they are known (61% strongly agree, 33% agree). Similarly, when considering the participation of someone close to them (friend, family member, colleague, etc), 91% of respondents strongly agreed or agreed that it was important that the person fully understood the known risks (56% strongly agree, 35% agree). 

### 2. Public views on a human challenge study with coronavirus taking place in the UK

**Figure 3.**
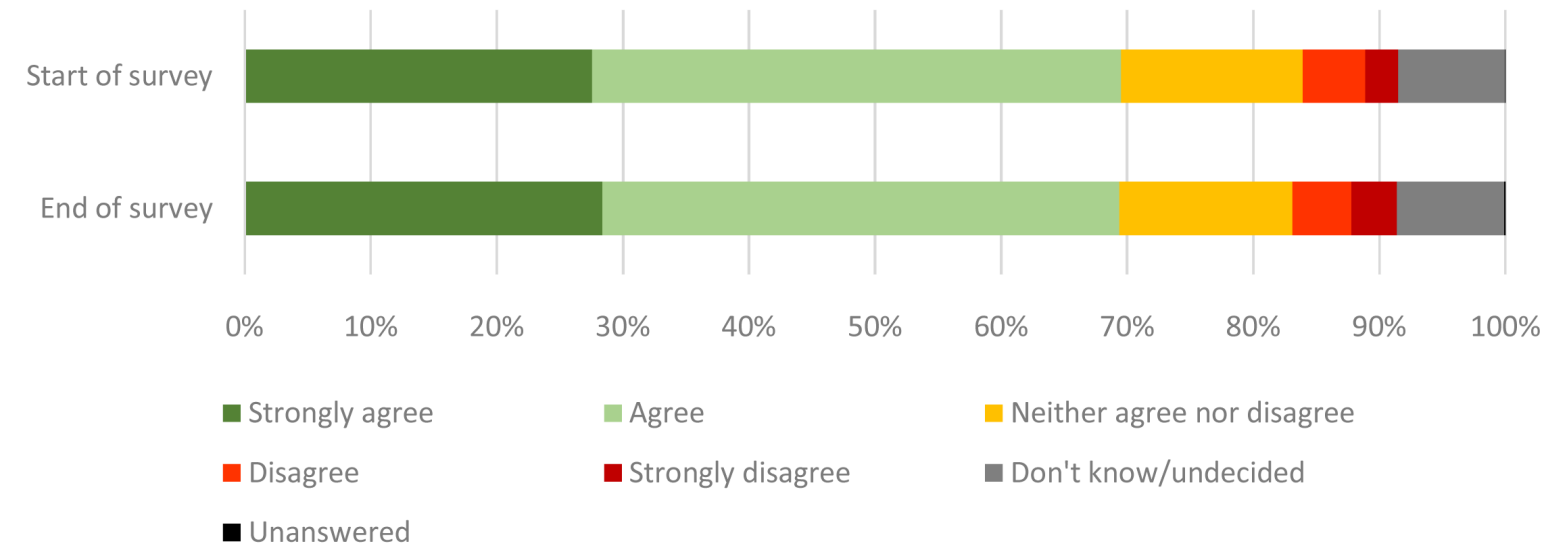
Agreement or disagreement with a human challenge study with coronavirus taking place in the UK. Answers the question “ **To what extent, if at all, do you agree or disagree that a human challenge study with coronavirus should take place in the UK?”** This question was asked at the start of the survey, after having watched the animation. As the survey content may have prompted considerations and/or reflections that might have changed an individual’s opinion of such a study, the question was asked again at the conclusion of the survey.

Survey respondents were asked to what extent they ‘agree or disagree that a human challenge study with coronavirus should take place in the UK’ ( Figure 3). This question was first asked at the start of the survey, following the animation. The survey provided a series of statements reflecting different opinions on a human challenge study with coronavirus. As these statements may have prompted considerations and/or reflections that might have changed an individual’s opinion of such a study, the question was asked again at the conclusion of the survey. On completion of the survey, 69% positively agreed with a study taking place (28% strongly agree, 41% agree).

***Trust***

The majority of survey respondents trust scientists and researchers to make the right decisions about a human challenge study ( Figure 2). Survey respondents were asked to what extent they trust researchers and the research ethics committee to make the right decision on whether a human challenge study with coronavirus should take place in the UK. A total of 73% of respondents strongly agreed or agreed with this statement (25% strongly agree, 47% agree). Respondents also trusted scientists to take safety precautions to make sure there would be no negative effects on the local community (31% strongly agree, 48% agree). Trust was also discussed in the focus groups.

*“There's a lot of distrust around… It's hard to know who to believe. When it comes to being sent certain articles by certain family members…Do you know what's real and what's not?” [FG3_18–30]*

***Acceptability***

Focus group discussants and survey respondents reflected on how acceptable they felt a human challenge study with coronavirus was including consideration of the value and purpose of such a study, ethical issues, balance of risks and benefits, and generalisability of findings across populations.

*“We are from a certain demographic… it might produce excellent results for that age group [younger and healthy] but would it produce results relevant for our group [older or less healthy]?” [FG6_31+]*
*“I think there is a level now where the ethics of what is allowed is very acceptable.” [FG6_31+]*
*“There seems to not be enough information to be able to accurately assess who would truly belong in the low risk group of people to infect deliberately, as the symptoms, short and longer term effects seem to me to be poorly understood at this time.” [Survey respondent]*

They also reflected on the unique nature of this pandemic, and how that impacted their thinking.

*“With Ebola it affected like a certain part of the world so I suppose I could ignore it if I wanted to, I didn't have to worry about it but with coronavirus, there's no way you can ignore it.” [FG3_18–30]*
*“We're virtually in a war situation, and in a war people do things that they wouldn't do normally, to help others … because we've never been in this sort of situation before where it's got so desperate, economically, psychologically, everything.” [FG9_31+]*

### 3. Perceived benefits of a human challenge study with coronavirus

A range of perceived benefits were identified for society, science and individuals.

***Urgency for a vaccine and treatment***

There was agreement that the current circumstances of the pandemic were characterised by an urgent need for the development of vaccines and treatments, not just for controlling the virus, but for providing hope to the general public.

*“So for me it's really all about time ... and speeding things up.” [FG9_31+] *
*“… uncertainties surrounding long-COVID but I think that increases … the importance of having human challenge study and … taking slightly more risks … to find a vaccine and find a treatment at an earlier stage” [FG7_18–30]*
“ *Until an effective vaccine has been developed, the health and wellbeing of everyone remains at risk... If a human challenge study can accelerate the delivery of an effective vaccine that mitigates all of these risks, it has to be pursued.” [Survey respondent]*

***Societal benefits***

Using human challenge studies was seen as contributing to the greater good in returning to ‘normal’, in order to reduce disruption to people’s lives, their wellbeing and the economy.

“ *We must explore all possible avenues to ensure we find a cure for this miserable virus before it costs more lives, more jobs, more misery” [Survey respondent].*

It was recognised that a human challenge study with coronavirus, undertaken under public scrutiny, presented an opportunity to educate the public about science, research and ethics. 

***Personal benefits***

People highlighted a range of personal gains to be had if they were to take part in a human challenge study. These included the financial incentive, the increased safety of being infected in a controlled environment, and motivations related to intrinsic personal rewards and experiences.

*“...you save up money from travel, parking and potential food costs so … if the money you're getting from the study outweighs all of that, I guess it will make economic sense for you to make a decision on that”. [FG4_18–30}.*
*“It would be convenient to be in a controlled setting … you feel like you are in a protected environment, a controlled and safe environment and were being monitored. So, if you're going to get the virus that's a safer way to get it”. [FG4_18–30]*
*“I'd actually love the isolation.” [Survey respondent]*

### 4. Perceived risks of a human challenge study

A range of perceived risks were also highlighted in relation to the study itself and the wider public. 

***Inducement***

A total of 73.6% of survey respondents agreed that, if payment were offered for taking part, people who were in a difficult financial situation might volunteer without fully considering the risks (22% strongly agree, 52% agree) ( Figure 2). These concerns were raised in the focus groups.

*“[referring to reimbursement] in an incredibly disadvantaged neighbourhood people will still take that money over the thought of their own safety… because if I saw a price tag that was like a grand, or even 500 pounds... I would think whoa, I am going to go for it.” [FG3_18–30] *

***Personal risks***

A range of risks to individuals from taking part were highlighted. These included risks to physical and mental health, and long-term implications such as impact on employment, mortgages, life or travel insurance. This includes the risk of long-term effects of COVID.

*“I think more than just the physical safety of participants we should also consider the mental health and well-being.” [FG8_18–30]*
*“There can be serious, deadly and long-term effects for people who develop the virus… and we can’t help very much with symptoms” [Survey respondent]*

***Study risks***

Participants felt that the study itself carried a number of risks relating to loss of public support if something goes wrong, potential transmission of the virus, and the study not achieving primary aims.

*“I think the other risk here is that, if it doesn't produce any anything useful or anything beneficial, then you might lose public support… or it's not actually speeded anything up … We've been promised all this and then it's not happened, kind of scenario, and that can be as damaging.” [FG5_31+]*

### 5. Taking part: perspectives of potential volunteers, loved ones and employees

Of those surveyed, 33% were willing to be purposefully infected with coronavirus as part of a human challenge study (10% strongly agree, 23% agree) ( Figure 2).

A total of 46% of survey respondents agreed that having to remain in isolation at a specialised medical unit for two weeks would influence their decision to take part (15% strongly agree, 31% agree) ( Figure 2). They were asked to identify potential problems related to staying in isolation at a specialised medical unit ( Table 4). In total, 87% of respondents identified one or more problems with taking part in the study (average of 3 problems per person). The most commonly identified problem was the negative impact on physical health (eg. experiencing short- or long-term symptoms of coronavirus) (44%) ( Table 4). Negative effects on mental health were identified as a potential problem by 32% of respondents.

**Table 4.** Potential problems identified by survey respondents with having to stay in a medical unit in isolation for 14 days. Answers the question: **Which, if any, of the following do you see as potential problems if you had to stay at a medical unit in isolation** **?** Other- animal was classified as a separate category based on analysis. Among other one off reasons, Other – unspecified included limited numbers for (1) would not take part, (2) not eligible – age, and (3) not eligible – health.

Problem	Percentage (Count)
It would negatively affect my mental health (e.g. I would get bored, stressed, lonely, anxious, angry, etc.)	32.3% (788)
It would negatively affect my fitness (e.g. my nutrition would be different, I couldn't exercise as usual, etc.)	25.7% (627)
It would negatively affect my physical health (e.g. I might experience short- or long-term symptoms of coronavirus)	43.5% (1063)
I would find it difficult to separate myself from others in my household (e.g. my partner, children, housemates, etc.)	35.5% (867)
I would find it difficult not being able to go outside	35.8% (874)
I have caring responsibilities and would find it difficult to find someone to cover those (e.g. I care for a dependent child, someone with disabilities, an elderly relative, neighbour, etc.)	19.8% (483)
It would negatively affect my social life (e.g. it would negatively impact my friendships, I would miss attending social and cultural events, etc.)	10.9% (266)
I would not be able to carry on working	26.2% (640)
I would have to use my annual leave	23.3% (569)
I would experience a loss of income	24.4% (595)
My studies or education would suffer	4.5% (109)
My employer would not be understanding or supportive	17.5% (428)
Other – animal responsibilities (eg. pets)	1.7% (41)
Other – unclassified	7.8% (190)
Don't know	3.1% (75)
Not applicable - I do not see any potential problems from staying at a medical unit in isolation	12.9% (316)

***Isolation***

Survey respondents highlighted it would be difficult to be away from household members (36%), or to not be able to go outside (36%), and negative effects on mental health (32%) and fitness (26%) ( Table 4).

Focus group discussants also expressed some concerns about the impact of isolation, such as the length of time involved and the level of freedom allowed.

*“If I'm feeling distressed, I have physical symptoms… I could imagine that a lot of people might have similar reactions if they're feeling isolated or depressed or alone for two weeks or just not really understanding what will happen to them” [FG4_18–30] *

***Remote working and impact on the human challenge study participant’s workplace***

The survey identified concerns relating to impact on work. Not being able to carry on working (26%), having to use annual leave (23%), loss of income (24%) and employers not being supportive or understanding (18%) were all identified as potential problems if they had to stay in isolation at a specialised medical unit ( Table 4). Within the focus groups, discussants felt that the ability to be able to work whilst taking part in a human challenge study would be an important consideration when volunteering. Employers discussed how an employees’ ability to take part in a human challenge study would very much be dependent on their role in the workplace, and how easily it would be to cover for them. A sense of altruism or duty was also present in this discussion.

*“if they're able to work throughout the study… there would be zero effect on productivity and output. it would have no effect on our team… should someone get seriously ill there'd be a lot of implications on morale [of the employer and wider team].” [FG3_18–30] *
*“I think it's important that there's sufficient publicity about the public value of people doing this … so that it's looked upon as a commendable activity and employers should not stand in the way of it.” [FG6_31+]*

***Comforts***

Discussants had suggestions about what might ensure the comfort of volunteers and reduce the negative impact of participating. This includes good care and support from research staff during and after the study, and the ability to communicate with friends and family. Features of the medical unit such as a television, Wi-Fi, exercise equipment and outside space were also viewed as important for being able to continue work (where relevant) and maintaining good mental and physical health.

*“I would like to see my family… access to doing something rather than just laying in bed all day so whether that's a film or a book… I would rather be isolated in my own home than being in a hospital” [FG7_18–30]*
*“Happy to isolate… if I can load up on games consoles and have some high speed internet ... then I'm fine.” [FG7_18–30] *

***Additional barriers to taking part***

Existing commitments, including caring for dependents (19.8%) and animals (1.7%), were identified as a potential problem by survey respondents if they had to stay in isolation at a specialised medical unit ( Table 4). Additional barriers raised include health issues and general distrust of unknown factors.

*“I would probably do it if it was like a really low dose or something, but I think if it was like one of the higher doses I would 100% be a no just because of all the unknowns.” [FG7_18–30] *
*“So potential participants in the challenge trials … may be swayed to think ‘I'm putting myself at risk and is there any point because people are getting reinfected’.” [FG9_31+]*
*“I have a rare blood group, and so am in demand by the blood service - if taking part would bar me from giving blood again, I'd need to balance participating against this restriction.” [Survey respondent]*

A total of 81% of survey respondents agreed that they would want to discuss the study with those there were close to before making a decision about whether to take part (40% strongly agree, 41% agree) ( Figure 2). Lack of support from family/friends was seen as a potential barrier to participation by focus group discussants.

***Acceptance of a volunteer’s decision***

Survey respondents were asked to consider someone close to them taking part in a human challenge study with coronavirus. They were asked if they would be supportive of a friend, immediate family member or work colleague taking part in a human challenge study with coronavirus ( Figure 2). There was 62–74% agreement that they would be supportive. They were also asked to indicate their agreement with the statements that taking part was either their close friend, immediate family member or work colleague’s decision to make. There was stronger agreement that it was an individual’s decision to make (78–90% agreement). There was lower agreement that people would be supportive of an immediate family member’s decision or that it was the family member’s decision to make than when considering the same scenario in reference to either a friend or colleague.

*“…the thought of my loved ones doing it has just made me realise what they would feel like if I did it” [Survey respondent]*
*“My son falls into the age category where … he could be used as a volunteer, and I'd be absolutely terrified … it's that fear of someone who you're very close to potentially putting their life at risk” [FG3_31+]*

In the focus groups, the older age groups and employers recognised the autonomy of others to make their own decisions and respected them for stepping up to make what was seen to be a brave and somewhat risky choice.

*“If it was someone that was close to me and I knew they were doing it for the right reasons. I'd be proud and I would be supportive”. [FG2_31+]*

***Additional concerns***

There were a wide range of additional concerns that would need to be addressed in order to inform decisions about volunteering. These included publicity, confidentiality, assurances about what would happen if something went wrong and intellectual property rights.

*“The press would be particularly interested in finding out everything that's going on with your sort of modern medical Big Brother household.” [FG6_31+] *
*“I would also want an assurance that should something happen or should there be any complications in terms of health that … I would be looked after or …. if anyone who was dependent would be looked after as well” [FG4_18–30]*
*“... who's gonna own the IP or whatever comes out at the end of the trial, how that's going to be shared? I would be funny about participating in one and putting my health on the line for a vaccine that would then be owned by a company who would make a lot of profit.” [FG1_18–30]*

## Discussion

This public consultation aimed to facilitate research transparency, in a time of heightened public interest and scrutiny of science, and to take a wide range of public concerns and opinions into consideration. Understanding public views and concerns is necessary to shape the design and communication of a study and ensure that research outputs are acceptable and translatable into real-world impacts.

Our research has shown similar findings to other stakeholder activities exploring the acceptability of human challenge studies ^26– 29^), although not all of these included members of the public as a stakeholder group. Our study went beyond exploring acceptability and identified practical considerations for teams designing human challenge studies. Recommendations are grouped into 1) Recruitment of volunteers; 2) Organisation of the study and 3) Involvement of the wider population. These were shared with the human challenge study team and were influential in ethics approval and study design ^20^.

### Acceptability of a human challenge study

The findings indicate that, at the time of the consultation (October 2020) there was a high degree of agreement that a human challenge study with coronavirus should take place. At the time of consulting, the UK was facing a second wave on COVID-19 and there were no approved vaccines. The data suggest a desire to change the situation, and vaccination was believed by many to be the only way out of the pandemic. Public awareness of long COVID was lower than it is today. In addition, there were misconceptions about human challenge studies, with some believing that these were a normal part of testing a vaccine. These factors are likely to change people’s perceptions on acceptability and the benefit to risk ratio.

Despite a high level of acceptability, this did not translate into a willingness to volunteer to take part. This suggests that while, at a societal level, the benefits outweighed the risks and concerns, this did not apply when considering the balance at an individual level. When talking about someone they were close to taking part, individuals described that they would be worried, albeit supportive, again suggesting that people viewed the risks as high despite feeling that it was important for progressing research.

### Use of the consultation to shape SARS-CoV-2 human challenge studies

As part of the consultation, the feedback was collated and shared with the SARS-CoV-2 human challenge study team in a report which detailed recommendations and the data that supported these recommendations. This report, along with feedback from the SARS-CoV-2 human challenge study team on whether, and how, the recommendations were addressed formed part of the Health Research Authority ethics submission. A thematic map ( Figure 1) identifies the main areas of consideration drawn from participant responses, supporting the identification of three main areas for which recommendations were made.

The consultation identified that potential volunteers would want time to discuss the study with others before making a final decision. This included family and friends as well as with employers. As a result, volunteers for the SARS-Cov-2 human challenge study are sent patient information and consent forms and then have a telephone call with a doctor/nurse prior to the first study visit. This gives volunteers time to consider the information, discuss it with family and friends, and ask any questions, before consent takes place. Because discussions with employers were identified as necessary, the study team created a document to provide information to employers about the anticipated impact on their employee’s work.

The impact of the study on mental wellbeing of volunteers was identified as a concern. We recommended that independent psychological and emotional support were provided to volunteers throughout the study, including follow-up once the isolation period ended. The SARS-Cov-2 human challenge study team began discussions with the Royal Free Hospital (where the study takes place) to establish access to specialist psychological support in the event that it was needed. We (the consultation team) do not know the outcomes of those discussions.

To our knowledge, this level of public consultation has not been done for other human challenge studies. The learnings have the potential to improve human challenge studies practice in three main areas: 1) messaging to potential study participants, 2) review of the protocol and organisation of the study, and 3) more broadly, making the study more inclusive and relevant. Based on the findings we recommend:

- Acceptability should be interpreted with context in mind, especially when considering the ethics of the human challenge studies at hand.
- Study materials should clearly indicate how human challenge studies are different to population level studies, in particular with regards to vaccine trials. Many members of the public have not heard of human challenge studies or may not understand them or aspects of the normal scientific methods of studying disease.
- Understanding the specific concerns that people have with the study environment is particularly important. Utilising public consultation and/or patient and public involvement to do this can be very effective, as it draws out considerations that study teams may not have considered.
- Equally as part of good practice in research, the concerns raised by the public should form part of the study review, with edits made as necessary. Where possible, how the information was reviewed and actions taken should be shared back to those involved.

### Strengths and limitations

The survey recruited a diverse sample of adults, largely matching United Kingdom demographics. However, the focus groups were less effective in recruiting this diversity and had limited numbers (n = 57). We were unable to recruit YouGov survey participants to the focus groups, due to YouGov strict policies on sharing identifiable information with clients. This, combined with short timescales for recruitment, limited the pool of potential participants for this stage of the study. Despite limitations with focus group numbers and diversity, the survey responses, including open text responses, largely mirror the results of the focus groups, supporting an ability to apply a level of generalisation to the findings.

We recognise that the context in which the study was carried out is important in understanding and interpreting the results. The results relating to the acceptability of a human challenge study are only generalisable in the fixed time period around when the study occurred. Opinions may have changed as the circumstances of the pandemic and the knowledge of the virus have changed.

## Conclusions

This study followed on from an initial consultation into the acceptability of a SARS-Cov-2 human challenge study in the UK ^19^. Our findings highlight a range of perceived potential benefits and risks of such a study. In line with WHO guidance on the key criteria for ethical acceptability ^30^, our findings informed the decision to proceed with a SARS-Cov-2 human challenge study in the UK, a world first, and informed the study design.

COVID-19 has brought unprecedented challenges and areas for potential advancement of science, medicine, and research. The results of this study highlight the value of public consultation in research, particularly in fields where public interest and scrutiny is high. Consultations should occur during initial consideration and design of studies. They should continue throughout the study to build transparency and trust in research, ensure the study remains acceptable to the public and ensure the outcomes have real-world impact.

## Data availability

### Underlying data

Zenodo: Public attitudes to a human challenge study with SARS-CoV-2: a mixed-methods study repository, https://doi.org/10.5281/zenodo.5733126 ^21^.

This project contains the following underlying data:

- 1D Survey answers.xlsx (Survey data (demographic information not included))

Due to confidentiality agreements, all supporting data cannot be made openly available. Consent was not given by focus group participants to publish transcripts in open access repositories. Demographic data gathered on participants involved in public consultations and study focus groups have been made available, as well as select anonymized quotes, as per approvals granted by the ethical review board Data with all direct and indirect identifiers removed can be made available through a sharing request. All sharing requests should be submitted to Prof. Helen Ward ( h.ward@imperial.ac.uk). Requests to allow/deny access will be handled within 20 working days of the original request.

### Extended data

Zenodo: Public attitudes to a human challenge study with SARS-CoV-2: a mixed-methods study repository, https://doi.org/10.5281/zenodo.5733126 ^21^.

This project contains the following extended data:

- 2A Focus Group Participant Information Sheet v4 14.10.20.pdf (Participant Information Sheet)
- 2B Focus Group Discussion guide v2.2 15.10.20.pdf (Focus Group Discussion guide)
- 1Ai Survey 01.10.20 v4.pdf (Survey)
- 1Aii Animation explaining the concept of a human challenge study with coronavirus.mp4 (Animation that survey participants were asked to view)
- Survey wrappers (invitations, participant information sheet and consent, end of survey messages)
  ○ 1Bi YouGov standard email invite to volunteer panel v1.2.pdf
  ○ 1Bii YouGov survey wrapper - Participant Information-Consent-End of Survey Message v3.2 06.10.20.pdf
  ○ 1Ci Qualtrics Invitation text v1.3 02.10.20.pdf
  ○ 1Cii Qualtrics Opportunity Advert - for VOICE + PERC website v1.3.pdf
  ○ 1Cii Qualtrics Participant Information Sheet v2.5 07.10.20.pdf
  ○ 1Ciii Qualtrics survey wrapper - Intro-Eligibility-Consent-End of Survey Messages v1.2.pdf
  ○ 2Ci Focus Group Invitation text v1.0 09.10.20.pdf
  ○ 2Cii Non -survey participant focus Group Invitation text v1.0 12.10.20.pdf
  ○ 2D Focus Group Registration text v1.0 09.10.20.pdf

Data are available under the terms of the Creative Commons Attribution 4.0 International license (CC-BY 4.0).
