# Supplementary material for: Public attitudes to a human challenge study with SARS-CoV-2: a mixed-methods study
Source: Wellcome Open Res. 2022 Feb 10;7:49. [Version 1] doi: 10.12688/wellcomeopenres.17516.1 (PMC8921687; doi:10.12688/wellcomeopenres.17516.1)
Supplement: Peer review history

# Peer review: Reviewer response for version 1

**DOI:** 10.21956/wellcomeopenres.19367.r48638

**Published:** 2022-03-14

**Authors:** Susan Bull

**Affiliations:** 1 The Ethox Centre, Nuffield Department of Population Health, University of Oxford, Oxford, UK; 2 Department of Psychological Medicine, Faculty of Medical and Health Sciences, The University of Auckland, Auckland, New Zealand

This is an interesting and well-written article reporting on a large scale study to inform the conduct of controversial research on a tight timeline. It is good to see this type of research being conducted and the results taken into account during design, review and implementation. Thank you for the opportunity to review - I have a few minor suggestions - the most substantive relating to clarifying Table 4.

 1. In the findings, the importance of clear communication about, and good understanding of, potential risks was discussed in the text and illustrated in a couple of places in Figure 2. The risks of SARS-CoV-2 CHIS also received substantial attention in academic and policy considerations about the ethics of such research. If there is more data it would be interesting to hear more about how personal research risks were perceived (there is just one sentence and a couple of quotes on page 11) and views about how best to communicate about them.

 2. Table 4 is described as being about potential problems associated with being in isolation for 14 days. However it includes the impact of infection on physical health (at line 3) which is not a consequence of isolation, but of exposure to infection. Some other aspects, such as impacts on mental health relating to anxiety, may also not be solely associated with isolation, but other perceived burdens of research participation (including exposure to infection). One approach to address this issue would be to revise the table title to encompass perceived risks/burdens/problems associated with research participation more generally (not just with isolation) - if doing this there may be more problems that it would be appropriate to incorporate. 

 3. On page 14 in the para beginning ‘The impact of the study on mental wellbeing’ the paragraph concludes by saying ‘We (the consultation team) do not know the outcomes of those discussions.’ Can clarity on this be achieved and reported, particularly as the authorship of this paper looks to include members of the SARS-Cov-2 human challenge study team?

 4. The first bullet point of recommendations on page 14 states ‘Acceptability should be interpreted with context in mind, especially when considering the ethics of the human challenge studies in hand’. The paragraph before the ‘Conclusions’ heading states ‘The results relating to the acceptability of a human challenge study are only generalizable in the fixed time period around when the study occurred.’ Taking these together – are you suggesting that the results of such acceptability studies should be considered specific to the context, study, and time period in which they are conducted? Alternatively – are you suggesting that some findings may be generalizable but others should be considered context-specific?

 5. The data availability section states that requests to allow/deny access will be handled within 20 working days. Do you mean decisions will be made within this timing (or are you anticipating there may be requests to deny access)?

Is the work clearly and accurately presented and does it cite the current literature?

Yes

If applicable, is the statistical analysis and its interpretation appropriate?

Yes

Are all the source data underlying the results available to ensure full reproducibility?

Partly

Is the study design appropriate and is the work technically sound?

Yes

Are the conclusions drawn adequately supported by the results?

Yes

Are sufficient details of methods and analysis provided to allow replication by others?

Yes

Reviewer Expertise:

Research ethics, including ethics of controlled human infection studies. Qualitative research.

I confirm that I have read this submission and believe that I have an appropriate level of expertise to confirm that it is of an acceptable scientific standard.

# Peer review: Reviewer response for version 1

**DOI:** 10.21956/wellcomeopenres.19367.r48637

**Published:** 2022-03-04

**Authors:** Dorcas Kamuya

**Affiliations:** 1 Health Systems and Research Ethics, KEMRI-Wellcome Trust Research Programme, Kilifi, Kenya

This is a great manuscript, which I enjoyed reading. The issue of conducting Human Infection Studies for SARs-COV2 met with significant debates globally, and this paper presents the views of potential participants. The use of mixed methods is particularly appropriate; the qualitative data adds depth to the quantitative data. I have no major comments as the paper is well written, just some minor comments which the authors can amend at their own discretion.

- Table 4 - was an attempt at prioritisation also done i.e. a question on which of the factors would be most important for resident stay?
- Interesting the last quote on IPR - as this is an area that we have not heard much about, but is gaining traction, particularly where industry participation is involved.
- It is interesting that the findings also support some of the work on this area e.g. wanting time to consult with others before deciding to join the study, and wondered whether this is unique to this type of study under the current COVID19 situation in the UK setting or is common with other types of studies as well. In our setting, it is certainly the case for HIS as well as other trials- that potential participants want to consult significant others, and the opinions of those others can influence their choice of participation.

Exciting to see the public participation and how that - as well as the social science study-  informed the design and roll out of the SARs-Cov2 challenge study in the UK.

Is the work clearly and accurately presented and does it cite the current literature?

Yes

If applicable, is the statistical analysis and its interpretation appropriate?

Yes

Are all the source data underlying the results available to ensure full reproducibility?

Yes

Is the study design appropriate and is the work technically sound?

Yes

Are the conclusions drawn adequately supported by the results?

Yes

Are sufficient details of methods and analysis provided to allow replication by others?

Yes

Reviewer Expertise:

Social science, research ethics

I confirm that I have read this submission and believe that I have an appropriate level of expertise to confirm that it is of an acceptable scientific standard.
